# Dyslipidemia, lipid-lowering agents and neuroendocrine neoplasms: new horizons

**DOI:** 10.1007/s12020-024-03767-7

**Published:** 2024-03-20

**Authors:** Roberta Modica, Anna La Salvia, Alessia Liccardi, Alessia Cozzolino, Antonella Di Sarno, Flaminia Russo, Annamaria Colao, Antongiulio Faggiano, on behalf of NIKE group

**Affiliations:** 1 Endocrinology, Diabetology and Andrology Unit, Department of Clinical Medicine and Surgery, Federico II University of Naples, 80131 Naples, Italy; 2 National Center for Drug Research and Evaluation, National Institute of Health (ISS), 00161 Rome, Italy; 3 Department of Experimental Medicine, Sapienza University of Rome, Viale Regina Elena 324, 00161 Rome, Italy; 4 Endocrinology Unit, Department of Clinical and Molecular Medicine, Sant’Andrea Hospital, ENETS Center of Excellence, Sapienza University of Rome, 00189 Rome, Italy; 5 UNESCO Chair, Education for Health and Sustainable Development, Federico II University, 80131 Naples, Italy

**Keywords:** Dyslipidemia, lipid-lowering agents, lipid metabolism, neuroendocrine neoplasm, neuroendocrine tumor, cancer therapy

## Abstract

**Purpose:**

Neuroendocrine neoplasms (NENs) are a heterogeneous group of malignancies originating from cells with a neuroendocrine phenotype. The complex relationship between lipid metabolism and cancer is gaining interest and a potential anti-cancer effect of lipid lowering agents is being considered. This review aims to discuss the current understanding and treatment of dyslipidaemia in NENs, focusing on the role of lipid lowering agents, including new therapeutic approaches, and future perspectives as possible tool in cancer prevention and tumor-growth control.

**Methods:**

We performed an electronic-based search using PubMed updated until December 2023, summarizing the available evidence both in basic and clinical research about lipid lowering agents in NENs.

**Results:**

Dyslipidemia is an important aspect to be considered in NENs management, although randomized studies specifically addressing this topic are lacking, unlike other cancer types. Available data mainly regard statins, and in vitro studies have demonstrated direct antitumor effects, including antiproliferative effects in some cancers, supporting possible pleiotropic effects also in NENs, but data remain conflicting. Ezetimibe, omega 3-fatty acids, fibrates and inhibitors of proprotein convertase subtilisin/kexin type 9 (PCSK9) may enhance the regulation of lipid homeostasis, as demonstrated in other cancers.

**Conclusions:**

Targeting dyslipidemia in NENs should be part of the multidisciplinary management and an integrated approach may be the best option for both metabolic and tumor control. Whether lipid lowering agents may directly contribute to tumor control remains to be confirmed with specific studies, focusing on association with other metabolic risk, disease stage and primary site.

## Introduction

Neuroendocrine neoplasms (NENs) are a heterogeneous group of malignancies originating from cells with a neuroendocrine phenotype, diffused in many organs and tissues, with variable aggressiveness and clinical behavior [1]. They mainly arise in the gastroenteropancreatic (GEP) tract and are mostly sporadic, but they can be associated with genetic syndromes [2]. NENs usually occur in adulthood or in elderly patients, usually show slow growing behavior, but metastases are often displayed already at diagnosis (40–76% of cases) and advanced stage negatively affects the prognosis [3]. Surgical treatment is preferred when feasible, but several therapeutic approaches used in diverse combinations and sequences are available, including somatostatin analogs, targeted therapies, peptide receptor radionuclide therapy, chemotherapy and liver directed therapies [4–6]. The therapeutic options are currently expanding, and new drugs are under development, with gaining interest towards functional pathways and molecular signatures in NENs aiming at tailored approaches [5, 7–9].

**Fig. 1:**
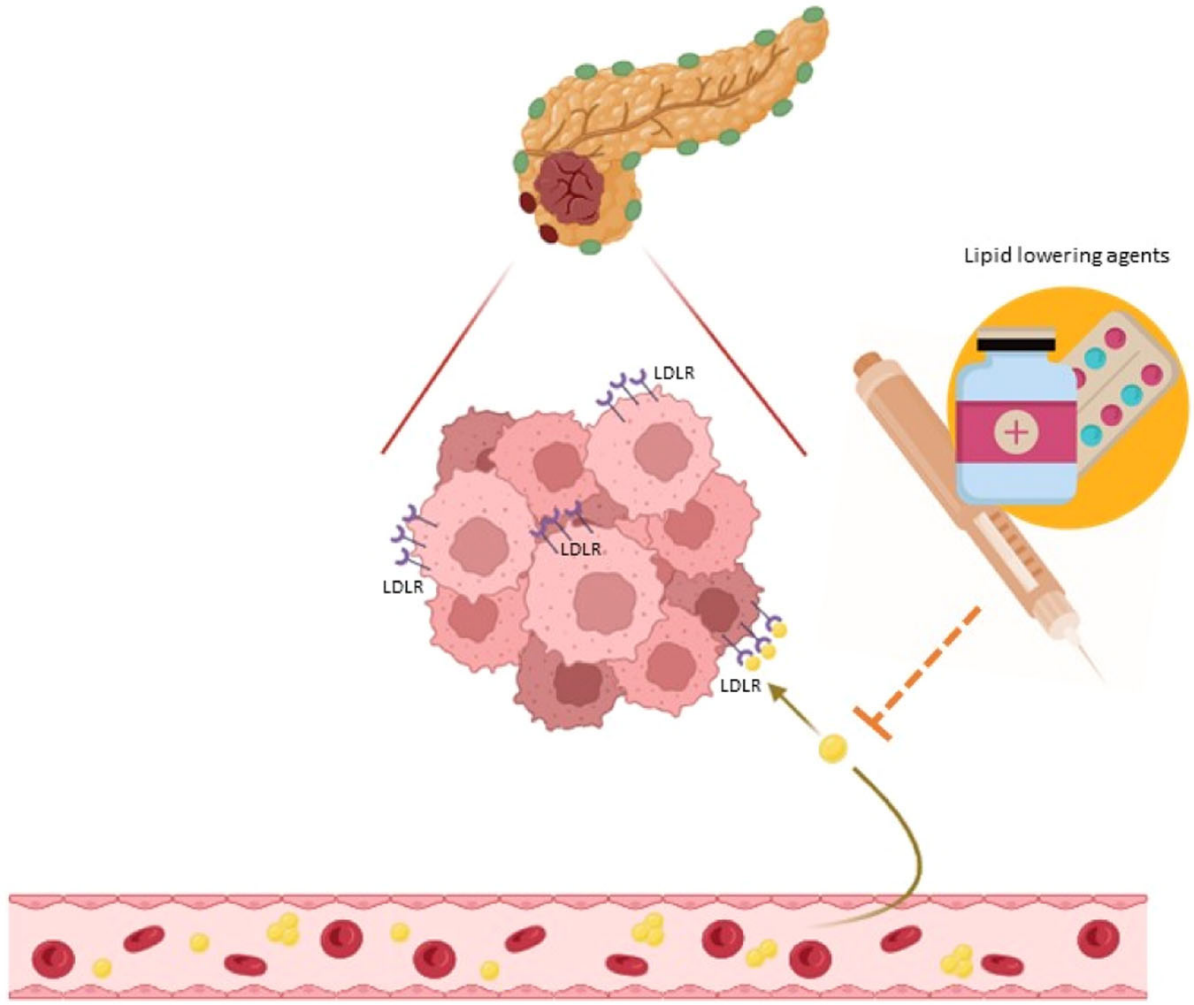
Interplay between lipids and pancreatic neuroendocrine tumor cells with effcts of oral lipid lowering agents. LDLR: low density lipoprotein receptor

The correlation between dysregulation of lipid homeostasis and cancer is acknowledged, with genetic and environmental factors modulating tumorigenesis [10–12]. In particular, reprogramming of lipid metabolism plays a significant role in survival and proliferation of cancer cells, as well as in metastatic spread [13]. The Cancer Genome Atlas project evaluated the mutational status and expression levels of all genes, including also those involved in cholesterol metabolism in different neoplastic tissues, supporting the role of upregulation of cholesterol synthesis in cancer development [14–16]. High circulating cholesterol levels have been considered as a risk factor for increased cancer occurrence, recurrence rates and treatment resistance [17]. Noteworthy in GEP-NENs type 2 diabetes and obesity have been reported as independent risk factors, highlighting the relevance of metabolic alterations in this cancer type [18]. Recently, it has been reported that low-density lipoprotein receptor (LDLR) is aberrantly expressed in numerous cancer histotypes, including those occurring in the gastrointestinal tract, in the liver, in the pancreas but also breast and lung carcinomas [19, 20]. LDLR has also been found to be involved in MAPK, NF-κB and PI3K/Akt signaling pathways, which affect cancer cells and their surrounding microenvironment [21]. Furthermore, elevated serum levels of low-density lipoprotein cholesterol (LDL-C) have been reported as a feature of endocrine and non-endocrine related tumors [22, 23]. The complex relationship between lipid metabolism and NENs is being thoroughly analysed, though molecular mechanisms remain far to be fully understood and the potential pleiotropic effects of lipid lowering agents needs in-depth analysis [24, 25]. A cross-sectional, case-control, observational study enrolling 109 grade 1 or 2 (G1/G2) GEP-NETs patients, compared with controls, reported that progressive and/or metastatic disease in GEP-NETs was associated with higher evidence of metabolic syndrome and non-alcoholic fatty liver disease [26]. In particular, LDL-C were significantly higher in GEP-NET patients than in the controls (*p* < 0.001), while high-density lipoprotein cholesterol (HDL-C) was lower (*p* = 0.034) [26]. Several molecular mechanisms have been proposed to explain the lipid-mediated cancer initiation and progression, including the induction of oxidative stress and the activation of oncogenic signaling pathways, but comprehensive data about NENs are lacking. Lipid lowering agents in NENs patients are currently used when dyslipidaemia is diagnosed, also as a side effect of anti-cancer treatments [24, 27]. In several cancer types, lipid lowering agents, statins in particular, have been supposed to have anticancer activities Figure. 1. It has been recently demonstrated that fasting may enhance the anti-tumor activity of several cholesterol biosynthesis inhibitors, including simvastatin, reducing Akt-Stat3 signaling and oxidative phosphorylation [28]. Interestingly Akt pathway is also involved in NENs, consequently, a role in cancer prevention and care for lipid lowering agents could be reasonably supposed [13, 29–32].

In this review we discuss the current understanding and future perspectives of dyslipidaemia in the context of NENs, focusing on the role of the mainly used lipid lowering agents, including new therapeutic approaches, as possible tool in cancer prevention and tumor-growth control. We performed an electronic-based search using PubMed updated until October 2023, summarizing the available evidence both in basic and clinical research about lipid lowering agents in NENs. The main studies carried out in the field of NENs, regarding both statins and other lipid lowering agents, with preclinical and clinical data, are summarized in Tables 1, 2, respectively.

**Table 1:** Statins and other lipid lowering agents in NENs: summary of preclinical available data

Study	Type	Cell line	Lipid lowering agent (s) tested	Results
Herrera-Martinez et al, [31] (30265346)	Preclinical	BON1 (pNET) QGP1 (pNET)	Atorvastatin Lovastatin Rosuvastatin Simvastatin	• Simvastatin/atorvastatin: decreased proliferation in BON1 cells; • All tested statins: decreased proliferation in QGP1 cells; • Simvastatin decreased migration capacity in BON1 cells and increased apoptosis in QGP1 cells; •Inhibition of phosphorylated AKT and ERK pathways
Vazquez-Borrego et al, [47] (31940630)	Preclinical	AtT20/GH3-cells PitNET	Atorvastatin Lovastatin Rosuvastatin Simvastatin	• All statins decreased AtT20-cell proliferation with stronger effects of simvastatin; • Simvastatin reduced cell viability and/or hormone secretion
Fliedner et al, [40] (24846270)	Preclinical	Mouse PHEO cells Mouse tumor tissue-derived cells (MTT)	Atorvastatin Lovastatin Rosuvastatin Fluvastatin, Simvastatin, Pravastatin,	• Higher anti-proliferative potency of simvastatin and fluvastatin compared to lovastatin; • Induction of apoptosis; • MTT cells more sensitive to statin treatment
Nölting et al, [51]	Preclinical	GOT (midgut NET) BON1 (pNET) H727 (lung NET) Mouse pheochromocytoma (MPC, MTT)	Lovastatin	• Synergic effect of lovastatin and the mTOR inhibitor everolimus in H727, MPC, MTT cell lines • Not additive effect in GOT and BON1 NET cell lines
Bai et al, [115] (28036293)	Preclinical	BON1 (pNET)	PCSK9	• PCSK9: promoted apoptosis and suppressed proliferation in pNENs
Mahoney et al, [116] (30626880)	Preclinical	K2 cells (SCLC) LU139 cell (SCLC) Tumor xenograft (SCLC)	NB-598 (inhibitor of SQLE)	• SCLC cell lines (in vitro) and SCLC mice xenografts (in vivo) are sensitive to NB-598

*MTT* mouse tumor tissue-derived cells, *NEN* neuroendocrine neoplasm, *NET* neuroendocrine tumor, *pNET* pancreatic NET, *pNEN* pancreatic neuroendocrine neoplasm, *PCSK9* proprotein convertase subtilisin/kexin type 9 inhibitors, *PitNET* pituitary NET, *SCLC* small cell lung cancer; SQLE (Squalene epoxidase)

**Table 2:** Statins and other lipid lowering agents in NENs: summary of clinical available data

Study	Type	Population	Lipid lowering agent(s) tested	Results
Herrera-Martinez et al, [31] (30265346)	Clinical	*N* = 181 (81 lung NET and 100 GEP NETs)	NA	• Higher proportion of patients treated with statins free of disease during the follow-up (*p* = 0.507); • No associations with other clinic-pathological variables
Awwad et al, [48] (35354139)	Clinical	*N* = 120 (pNET)	NA	• Increased mortality in patients receiving statins after curative intended surgery
Sahi et al, [54] (22683172)	Clinical	*N* = 50 (MCC)	Atorvastatin Lovastatin Rosuvastatin Fluvastatin, Simvastatin, Pravastatin	• Standardized incidence ratio (SIR) for MCC of 1.94 in ages 60–74 and a SIR of 3.16 in ages <60 years among statin users compared to statin nonusers
Bai et al, [115] (28036293)	Clinical	*N* = 205 (22 lung NET and 183 GEP NENs)	NA	• The average levels of serum lipid in NENs patients were significantly lower than the healthy controls • No significant correlation between survival and level of LDL-C (*p* = *0.08*)

*GEP* gastroenteropancreatic, *LDL-C* low-density lipoprotein cholesterol, *MCC* Merkel cell carcinoma, *NA* not available, *NEN* neuroendocrine neoplasm, *NET* neuroendocrine tumor, *pNET* pancreatic NET, *pNEN* pancreatic neuroendocrine neoplasm, *SIR* standardized incidence ratio

## Statins and NEN

Statins lead worldwide consumption of lipid-lowering drugs, whose prescriptions is significantly growing due to the increasing incidence of dyslipidemias also in cancer patients [33]. Statins are commonly used drugs in the therapeutic arsenal for patients with metabolic syndrome or type 2 diabetes mellitus, and specifically they are prescribed in clinical practice to treat hyperlipidemia and in cardiovascular or coronary heart diseases, as well as in both primary and secondary prevention [34]. Statins inhibit the enzyme 3-hydroxy-3-methylglutaryl-coenzyme A reductase, affecting the rate-limiting step in cholesterol synthesis. Besides their cholesterol-lowering effects, statins have been demonstrated exerting a wide range of pleiotropic effects, including immunomodulatory, anti-oxidant and anti-inflammatory effects acting through cholesterol-dependent and -independent mechanisms [34, 35]. NETs exploit a variety of complex signaling molecular pathways for their development, growth and survival, including the phosphatidylinositol 3-kinase-Akt (PI3K-Akt) and the Ras/Raf/mitogen-activated protein kinase (MEK)/extracellular signal-regulated kinase (ERK), which could also represent a therapeutic target for statins [36, 37]. Despite molecular mechanisms underlying anticancer effects of statins in NETs are far to be fully elucidated, is it possible to assume a similarity with other cancers due to common signaling pathways, as in breast cancer cells, where simvastatin has been demonstrated to activate the ERK1/2 and Akt pathways, suppressing autophagy and promoting cell death [38]. Likewise NETs share signaling pathways with renal cell carcinoma (RCC) and interestingly in RCC statins are known to inhibit the phosphorylation of AKT, mammalian target of rapamycin (mTOR), and ERK reducing cells motility [39].

In the last ten years, several studies have also related the use of statins with antineoplastic properties in different tumors [40–44]. A meta-analysis evaluating several types of cancer revealed that the use of statins seems to be beneficial for overall survival and cancer-specific survival [45]. In vitro studies have demonstrated that statins exert direct antitumor effects, including antiproliferative effects, inhibition of migration and invasion, proapoptotic actions, and cancer-stem cells inhibition [42, 43, 46].

Recently, based on the potential association among type 2 diabetes mellitus, metabolic syndrome, and cancer, Herrera-Martinez and coworkers explored this association in a population of 181 NETs, among which 81 were lung carcinoids and 100 were GEP NETs and analyzed the use of statins in these cohorts, exploring their putative relationship with clinical and histological characteristics [31]. The results of the study showed no clinical, histological, or molecular variable associated with the presence of hyperlipidemia, with a higher proportion of patients treated with statins free of disease during the follow-up. Conversely, none of the other clinical, histological, or evolution parameters were associated with the use of statins. Moreover, the authors investigated the potential in vitro antitumoral effects of different statins (namely, atorvastatin, lovastatin, rosuvastatin, and simvastatin) in two different NET-cell models: BON1 and QGP1 cell lines and found that effects of statins on proliferation rate depended on the statin and cell types, and time. Specifically, only simvastatin and atorvastatin decreased proliferation in BON1 cells, whereas all statins decreased proliferation rate in QGP1 cells. Simvastatin decreased migration capacity in BON1 cells and increased apoptosis in QGP1 cells. Furthermore, they observed an inhibition of phosphorylated AKT and ERK pathways, whose exerted role in NETs’ pathogenesis is well known, after treating cells with simvastatin, which reveals the AMPK-dependent and -independent effects of statins in NET cells [31].

Another study from the same group explored the effects of statins in cell proliferation/viability, hormone secretion, and signaling pathways in tumor cells from corticotropinomas, somatotropinomas, pituitary tumors (PitNET), PitNET cell-lines (AtT20/GH3-cells) [47]. The results of this study showed that all statins decreased AtT20-cell proliferation with stronger effects of simvastatin. Indeed, simvastatin reduced cell viability and/or hormone secretion in all PitNETs subtypes and cell-lines, unveiling direct antitumor effects of simvastatin on PitNET-cells and suggesting these compounds as a possible tool to treat this kind of NEN [47].

On the other hand, a recent study by Awwad et al. evaluated retrospectively the influence of metabolic syndrome in 120 patients with curative intended resection of pancreas NETs (pNETs) on overall survival, recurrence-free survival, and outcome after recurrence and, analyzing single metabolic syndrome components, found that IFG/TDM2, hypertension, and use of statins were associated with an increased hazard for mortality in pNET patients after curative intended surgery [48]. It is conceivable that this finding could reflect the increased mortality of the underlying disorder leading to statin use rather than a direct drug-mediated effect on the course of the neoplastic disease.

Previously, statins have been reported to exert their anti-neoplastic effect amongst others by MAPK pathway inhibition [49, 50]. The MAPK pathway plays a role in numerous aggressive tumors and, specifically, it has been associated with a subgroup of malignant pheochromocytomas and paragangliomas, including K-RAS-, RET-, NF1- and SDHB-mutated tumors, thus suggesting that inhibition by statin treatment could be beneficial in these settings [40, 41].

Based on this assumption, Fliender et al. assessed the anti-proliferative effect of different statins on mouse PHEO cells (MPC) and the more aggressive mouse tumor tissue-derived cells (MTT). The results of their study showed a higher anti-proliferative potency of simvastatin and fluvastatin compared to lovastatin, with the more aggressive MTT cells the more sensitive to statin treatment, suggesting that more aggressive cells may be more receptive to the anti-proliferative effects of statins. Moreover, the authors found increased levels of CASP-3 and PARP cleavage, confirming induction of apoptosis following the treatment and spontaneous migration of MPC and MTT was significantly inhibited within 24 h, thus unveiling lipophilic statins as a promising therapeutic option for treatment of aggressive human paragangliomas [40].

Of note, also the combination of statins and the mTOR inhibitor everolims has been tested in vitro [51]. In this preclinical study, the activity of lovastatin plus everolimus has been investigated in several cancer cell lines, including human midgut (GOT), pancreatic (BON1), and pulmonary (H727) NET, hepatocellular carcinoma (HepG2, Huh7) cell lines, and mouse pheochromocytoma (MPC, MTT) cell lines. The authors a synergic effect of the two agents, both administered at clinically relevant doses, in pulmonary NET, mouse pheochromocytoma and hepatocellular carcinoma cell lines, whereas this additive activity was not confirmed in midgut or pancreatic NET cells.

With regard to statins’ immunomodulatory effect, increasing evidence has suggested that, beyond promoting atherosclerotic plaque stability, it also hinders the host antitumor immune response, therefore potentially increasing cancer risk in a subset of patients, especially in those tumors of viral origin. In particular, the immunosuppressive actions of statin therapy have been linked to increasing Merkel cell carcinoma (MCC) risk and progression [52–54].

MCC is a rare and severe cutaneous neuroendocrine malignancy with a tendency to early and frequent locoregional-to-systemic metastasis and relapses. Merkel cell polyomavirus (MCPyV), a small polyomavirus with double-stranded DNA, has been recognized as a new etiological pathway leading to MCC, confirmed by the integration and truncation of large T (LT) viral antigen in MCC cells [55, 56]. A Finnish study evaluating a cohort of 224 715 male and 230 220 female statin users during 1994–2007, identified from the Prescription Register of the National Social Insurance Institution, found a standardized incidence ratio (SIR) for MCC of 1.94 in ages 60–74 and a SIR of 3.16 in ages <60 years among statin users compared to statin nonusers, suggesting a role of statins in increasing the risk of MCC in atypically younger individuals, comparable to that observed in patients with immunocompromising states [54].

Statins’ immunomodulatory effects are mediated by several mechanisms including inhibition of natural killer cell cytotoxicity and degranulation, decrease of dendritic cell function, increase of the numbers and functionality of peripheral regulatory T cells (Tregs), whose decrease has been recently linked to the reduction of T-cell responses in MCC [52, 53]. Thus, statin therapy might in part explain the increasing incidence of MCC and result in poorer MCC-specific survival.

Overall, statins’ role on cancer risk is still controversial. Nevertheless, published evidence of their anti-neoplastic effects in NETs, the lack of a satisfactory antineoplastic therapy in advanced NETs, together with statins’ low-cost, commercial availability, safe profile and large experience in clinical use, suggest further exploration of their therapeutic potential for patients with NETs.

## Other lipid lowering agents and NENs

Other lipid lowering agents mainly include ezetimibe, omega 3-fatty acids, fibrates and inhibitors of proprotein convertase subtilisin/kexin type 9 (PCSK9). Ezetimibe, is an oral lipid lowering drug usually taken after statins or in combination with them; it blocks Niemann–Pick C1-like 1 protein (NPC1L1), a human sterol transport protein expressed both on the apical side of jejunal enterocytes and on hepatobiliary tract [57]. Through its action ezetimibe both inhibits intestinal cholesterol absorption and decreases biliary cholesterol secretion, lowering LDL-C and reducing the occurrence of cardiovascular events. Ezetimibe has been associated with increased cancer risk, and a recent meta analysis mainly identified a possible increase in intestine cancer risk and a trend of increasing risk of breast cancer [58]. Nevertheless, other studies are inconsistent, supporting ezetimibe as a potential anti-cancer drug, thus the potential harm of ezetimibe remains debated [59]. Nowadays data regarding ezetimibe and NENs are not available yet, and the supposed mechanism of ezetimibe as a tumor inhibitor is not yet completely understood. One of the mechanisms proposed to explain the role of ezetimibe in cancer development and growth is that it may be able to inhibit CD31 (platelet endothelial cell adhesion molecule and increase TSP-1 and SMA) (smooth muscle actin, a perivascular cell marker) expression inhibiting the angiogenesis, promoting apoptosis and preventing cell proliferation. These observations could support an investigation in NENs, which usually are highly vascular cancers [60].

Omega 3-fatty acids and fibrates are approved in case of persistent hypertriglyceridemia despite an appropriate diet [61]. Fatty acids (FAs) are a large group of aliphatic monocarboxylic acids formed by long chains with an even number of carbon atoms without ramifications and cyclical forms (saturated fats, without double bonds between carbons and unsaturated fats, with double bonds between carbons). They lower plasma levels of very-low density lipoproteins (VLDL) – and consequently of triglycerides - by increasing fatty acids oxidation, therefore decreasing hepatic lipogenesis; they also seem to improve the clearance of chylomicrons [62]. One of the most studied FAs in the search for anti-cancer drugs from food sources is docosahexaenoic acid (DHA). DHA is a type of omega-3 long-chain polyunsaturated FA with role in the prevention of cardiovascular diseases and premature retinopathy, promoting anti-inflammatory action and anticancer activity [63–66]. In vitro studies investigated DHA anti-cancer activity on breast, lung, colorectal, prostate and blood cancer cell lines [67–77]. These results led to human study and clinical trials especially in colonic, breast and hematological cancer population [78–84]. Particularly, its beneficial effect was observed as chemotherapy coadjuvant treatment helping to better tolerate this intensive therapy. Some enzymes involved in FAs and cholesterol’s synthesis have been suggested as prognostic biomarkers in common cancer types, including prostate and breast cancer, in which FA synthase (FASN), a key lipogenic enzyme catalysing the terminal steps in FA biogenesis, was found to be upregulated [85]. If similar findings could be demonstrated in NENs, inhibition of FASN could be tested to prove tumor growth inhibition. However, data in NEN regarding FAs therapy as anti-cancer drug are currently lacking. Nevertheless, in light of the available data on other diverse cancer types it could be supposed a beneficial effect also in NEN patients affected by poorly differentiated and metastatic disease requiring chemotherapy. Regarding fibrate, it mainly reduces triglycerides, variably lowering LDL-C. It explicates its action through peroxisome proliferator-activated receptor-α (PPAR-α), a transcription factor which, when activated, increases lipoprotein lipolysis and hepatic fatty-acid uptake [86]. It has been demonstrated in rats that oral administration of ciprofibrate, for 2 or more weeks at doses of 20 mg/kg/day or more caused hypertrophy and increased eosinophilia of the oxyntic cells in the gastric mucosa. Hyperplasia of the neuroendocrine cells occurred after prolonged administration for more than 2 months and after 2 years the formation of gastric NET was documented. Importantly the formation of gastric NET following ciprofibrate administration was not confirmed in mice and marmoset thus supporting that this cancer specifically arises in species such as the rat when significant gastric antisecretory activity occurs [87]. Promising therapeutic drugs for the treatment of hypercholesterolemia and associated cardiovascular disorders are the inhibitors of PCSK9, namely evolocumab, alirocumab, inclisirian and bococizumab. The latter was withdrawn from development due to conflicting data about its efficacy. PCSK9 inhibitors are recommended in primary prophylactic treatment for cardiovascular disease when the lipid target in accordance with cardiovascular risk is not achieved during treatment with statins at the highest tolerated dose or in secondary prevention to a cardiovascular event [61]. Regulation of neuronal apoptosis and modification of plasma lipid homeostasis via LDLR expression, both intracellular and extracellular, are PCSK9’s two primary biological activities [88, 89]. Hepatic LDLR are destroyed thanks to PCSK9 action, causing an increase in LDL-C levels. PCSK9 inhibition, therefore, significantly reduces plasmatic LDL-C by improving hepatocytes capacity to re-move it from the bloodstream [90]. PCSK9 can also interact with other LDLR-like family members, particularly the very-low density lipoproteins receptor (VLDLR) and apoER2 [91]. Importantly, anti-cancer and immune-stimulating characteristics of PCSK9 inhibitors have emerged from recent preclinical and clinical findings. In some studies, using lipid lowering medications, a link between low levels of LDL-C and incident cancer risk has been shown, however contrasting data emerged about the use of these drugs [92–94]. In addition to its function in cholesterol metabolism, PCSK9 is also involved in the cell cycle, inflammation, and apoptosis and is overexpressed in both differentiating cells and numerous human cancer cell lines [95–98]. Indeed, Neural apoptosis-regulated convertase 1 (NARC-1) is encoded by the PCSK9 gene and is involved in the propagation of apoptotic signaling in neurons [99]. Regarding this, Bath et al. speculated that decreased PCSK9 expression could promote hepatocellular carcinoma (HCC) [100]. This study enrolled 39 patients with HCC, their liver tissue samples were analysed by immunostaining for PCSK9 after surgery revealing an increased LDLR expression together with a decreased PCSK9 expression in HCC cells. Moreover, with this phenotype HCC cells can provide for cholesterol intake, hence PCSK9 inhibition may be useful in reducing the metabolism of HCC and, consequently, the growth potential of the disease. Importantly the liver is the main metastatic site of NENs, but analysis of PCSK9 expression in neuroendocrine liver cells is not currently available [101]. On the contrary PCSK9 expression was upregulated in colon cancer cells compared with non‐tumor cells and correlated with the degree of tumor invasiveness, indeed PCSK9 appeared to mediate MIF and lactate levels to influence tumor-associated macrophage polarization towards activated or anti-infammatory phenotype that promote tumor growth [102]. A Chinese study found that PCSK9 participates in cell growth and cell cycle of HCC, being able to reduce apoptosis by interacting with GSTP1 and inhibiting the JNK signaling pathway [103]. Similarly, Demidyuk et al. analysed human lung cancer samples to identify PCs genes comparing to no-tumor tissue samples. In this study, in tumor tissue a statistically significant reduction of PCSK9 mRNA levels (PCSK2, PCSK5, PCSK7, PCSK9) and an increase in PCSK1 mRNA expression was demonstrated [104]. More recently a pilot study has investigated the prognostic role of PCSK9 in patients with non-small lung cancer (NSCLC), and they found that in patients with advanced, previously treated NSCLC, serum PCSK9 levels greater than 95 ng/mL at the second cycle of nivolumab therapy was an independent predictor of decreased overall survival (OS) [105]. For this result, the authors suggested to follow up patients with advanced NSCLC evaluating serum PCSK9 [105]. Likewise, PCSK9 systemic level was found higher in stage III lobular or ductal breast cancer patients than in patients with benign disease or less aggressive stage [106]. The lack of reliable prognostic marker in NENs could lead to consider the evaluation of PCSK9 as a possible tool in the therapeutic management [107]. Furthermore, inflammation is also involved in the relationship between cholesterol and cancer. Indeed, LDLR increases after a 24-hour stimulation with lipopolysaccharide (LPS) of HCC cell leading to a higher cholesterol uptake that may contribute to cancer cell survival as already proposed [108]. More data are available regarding the PCSK9 role in the oncological immune tolerance [109]. Indeed, PCSK9 prevents the recycling of major histocompatibility complex type I (MHCI) to the cell surface promoting intratumoral infiltration of cytotoxic lymphocytes [110, 111]. The same mechanism is used to promote lysosomal degradation of CD81 and CD36, as well as LDLR already mentioned [112]. Hence, PCSK9 inhibitors may develop peripheral immunological tolerance against tumor cells improving T-lymphocyte identification. In preclinical study, in neuroglioma and NSCLC knockdown of PCSK9 gene determinants cancer apoptosis using the caspase‐3 and XIAP/p‐Akt pathways and in melanoma-bearing mice PCSK9 gene silencing considerably boosts the response to immune checkpoint inhibitors (ICIs) [113, 114]. Considering the elevated risk to develop atherosclerotic plaques and consequently atherosclerotic cardiovascular disease in cancer patients who underwent ICIs therapy, PCSK9 inhibitors may increase their OS by reducing LDL-C. In the neuroendocrine field PCSK9 role has been mildly analysed and the results are not in line with previous observations. A retrospective study enrolled 205 NENs showing that mean levels of total triglyceride, total cholesterol, HDL-C, and LDL-C were all significantly lower in NENs patients than in healthy controls. Moreover, low LDL-C level was significantly correlated with survival rate and median OS, namely patients with LDL-C > 159 mg/dl appear to have a better OS than patients with LDL-C < 101. Hence, lowering LDL-C may be inappropriate in this disease. In addition, tissue samples of pNENs were analysed performing an immunohistochemistry assay and the authors found out that PCSK9 is a direct target of miR-224. The increase of miR-224 causes the decrease of PCSK9 and this could promote apoptosis and suppress proliferation, invasion of BON-1 cells in pNENs (cell line frequently used in pNEN model) [115]. In conclusion, PCSK9 inhibitors may be considered in human trials to control cholesterol metabolism verifying who could benefit from this therapy, supporting the possible role of these drugs in combination with avelumab that is the only ICI approved in NENs for the treatment of MCC.

Squalene epoxidase (SQLE), a cholesterol biosynthetic pathway enzyme, has been recently identified as a potential therapeutic target in NENs. Indeed, in neuroendocrine cell lines a sensitivity to NB-598, a known inhibitor of SQLE, has been demonstrated with cell growth defects and animal models confirmed this finding in vivo [116]. These observations shed a light on novel potential therapeutic targets, that need to be tested in clinical trial to confirm their efficacy.

## Conclusions and future directions

Tailored therapy is the current aim in NENs, to obtain tumor mass reduction or stabilization, improvement of patients’ symptoms and quality of life, as well as survival rates’ growth, thus a multidisciplinary approach is essential [5, 117, 118]. In this light, a better understanding of the interactions between lipid lowering agents and NENs’ onset and progression could lead to more effective and customized treatments for patients. Beneficial effects of lipid lowering agents, especially statins, have been documented in several diseases including chronic kidney disease and chronic inflammatory disease, but confounding factors when evaluating their anti-cancer effect may exist [119]. Randomized controlled trials regarding the effect of lipid lowering agents in NENs are lacking as the majority of reported data mainly concern their relationship with malignancy in general. Consequently, robust data clarifying the relationship of dyslipidemia and lipid-lowering agents with NENs are currently difficult to obtain. Metabolic alterations including dyslipidemia have been shown to augment the risk of cancer and to worsen its prognosis in NENs [26]. Specifically, dyslipidemia is a strong predictor of cardiovascular disease, which may become, as in other cancers, the leading contributor to morbidity and mortality with ageing also in NENs, that usually have an indolent course [120, 121]. Current indications for lipid lowering agents in NENs only indirectly target tumor growth, through decreasing associated risks in both primary and secondary prevention of cardiovascular risk. The current challenge remains the assessment of tumor growth control with lipid lowering agents, which could expand their use with new therapeutic indications. Nonetheless, early detection and treatment of dyslipidemia should be integrated in the multidisciplinary NENs patients’ management. A role of lipid lowering agents in cancer prevention is difficult to assess, also due to the late diagnosis in NENs, but a close lipid profile control is advisable irrespective of disease stage, since beneficial effects on long-term survival could be obtained. The future looks bright for promising treatments that improve lipid profile and further studies focused on association with other metabolic risk, disease stage and primary site are needed to identify the best lipid lowering agent and the optimal timing of therapy administration. Further investigations should be designed also with the aim to clarify the conflicting data emerging within each lipid lowering drug and to understand if novel biomarker as PCSK9 or FASN can be recognized and usefully integrated in the therapeutic management. Prospective randomized study, also with combined therapies with new agents, may be the key to identify individual approaches integrating both lipid and tumor control.
